# The Prognostic Significance of *IRF8* Transcripts in Adult Patients with Acute Myeloid Leukemia

**DOI:** 10.1371/journal.pone.0070812

**Published:** 2013-08-14

**Authors:** Era L. Pogosova-Agadjanyan, Kenneth J. Kopecky, Fabiana Ostronoff, Frederick R. Appelbaum, John Godwin, Hana Lee, Alan F. List, Jennifer J. May, Vivian G. Oehler, Steve Petersdorf, Galina L. Pogosov, Jerald P. Radich, Cheryl L. Willman, Soheil Meshinchi, Derek L. Stirewalt

**Affiliations:** 1 Clinical Research Division, Fred Hutchinson Cancer Research Center, Seattle, Washington, United States of America; 2 Southwest Oncology Group Statistical Center, Fred Hutchinson Cancer Research Center, Seattle, Washington, United States of America; 3 Department of Oncology, University of Washington, Seattle, Washington, United States of America; 4 Providence Cancer Center Group, Earle A. Chiles Research Institute, Portland, Oregon, United States of America; 5 H. Lee Moffitt Cancer Center, Tampa, Florida, United States of America; 6 Seattle Genetics, Inc., Bothell, Washington, United States of America; 7 University of New Mexico Cancer Research and Treatment Center, University of New Mexico, Albuquerque, New Mexico, United States of America; UT MD Anderson Cancer Center, United States of America

## Abstract

Interferon regulatory factor 8 (*IRF8*) is a transcription factor that plays a critical role in normal hematopoiesis, such that disruption of IRF8 activity promotes leukemogenesis. We and others have identified aberrant expression of *IRF8* transcripts, including novel splice variants, in acute myeloid leukemia (AML), but studies have not investigated the prognostic significance of these transcripts. Therefore, we developed and optimized quantitative expression assays for both, the wild type, or the reference sequence (WT-*IRF8)* and novel splice variants (SV-*IRF8*). These assays were used to quantify IRF8 transcript levels in 194 adult patients with AML, and multivariate analyses investigated the prognostic significance of these expression levels. After adjusting for known prognostic factors, expression levels of WT- or SV-*IRF8* transcripts were not significantly associated with complete responses or overall survival. However, increased expression of WT-*IRF8* was associated with decreased relapse-free survival (RFS) in both univariate (P = 0.010) and multivariate (P = 0.019) analyses. Similarly, increased expression of SV-*IRF8* was associated with a decreased RFS (univariate, P = 0.026 and multivariate, P = 0.021). These studies show for the first time that WT-*IRF8* and SV-*IRF8* are independent adverse prognostic factors for patients with AML. Additional studies are planned to examine the prognostic significance of *IRF8* transcripts in other populations of AML patients.

## Introduction

Interferon regulatory factor 8 (*IRF8*) is a tightly regulated transcription factor [1], [2]. Modest changes in IRF8 activity help to determine hematopoietic lineage, and disruptions in this activity detrimentally impacts normal hematopoiesis [1], [2]. For example, loss of IRF8 function disproportionately promotes the development of granulocytes and blocks normal monocytic differentiation. Conversely, constitutive expression of IRF8 promotes the development of monocytic/dendritic-like cells and blocks normal granulocytic differentiation [3]–[5]. Over time, IRF8-related dysregulation may promote malignant transformation, and in fact, inactivation of IRF8 in mice models causes a myeloproliferative syndrome that progresses to an AML-like disease [6]–[8].

We and others have shown that aging hematopoietic stem cells and primary leukemic blasts frequently display aberrant expression of the wild type reference sequence for *IRF8* (WT-*IRF8*, NM_144701) [9]–[15]. In addition, we have recently identified novel splice variants for *IRF8* (SV-*IRF8*) in malignant cell lines and leukemic blasts from AML patients, which are subsequently described in this manuscript. Although the functional significance of the splice variants remains unknown, these SV-*IRF8* transcripts appear to be the result of aberrant hypermethylation of the normal *IRF8* promoter, and as such, may not be subject to the normal regulatory factors that control WT-*IRF8* expression (unpublished data).

The available data indicate that normal IRF8 activity is essential for a healthy hematopoietic system and that disruption of this activity may promote leukemogenesis. Despite this data, studies have not examined the prognostic significance of *IRF8* transcripts in AML or other hematopoietic malignancies. Therefore, we investigated the prognostic significance of *IRF8* transcripts in a large cohort of adult patients with AML who were treated on Southwest Oncology Group (SWOG) protocols. These studies showed for the first time that *IRF8* transcripts were independent adverse prognostic biomarkers for adult patients with AML.

## Materials and Methods

### Ethics Statement

All participants provided written informed consent in compliance with the Declaration of Helsinki to permit the use of their samples for research. Documentation of consent was provided to and maintained by SWOG, and only samples from consented subjects were included in the studies. All *IRF8* studies were conducted with approval of the Fred Hutchinson Cancer Research Center's Institution Review Board, which oversees the ethical conduct of research at this center.

### Patient material

AML samples used for development of WT-*IRF8* and SV-*IRF8* assays were obtained from the Fred Hutchinson Cancer Research Center and the University of Washington Leukemia Repository. Peripheral blood from normal donors was obtained under FHCRC IRB-approved protocol. Cell lines were purchased from ATCC (Manassas, VA). RNA from diagnostic bone marrow (BM, N = 155) or peripheral blood (PB, N = 39) samples were obtained from 194 previously untreated AML patients who received cytarabine (Ara-C) and daunorubicin (DNR)-based induction as part of SWOG-9031, SWOG-9126, SWOG-9333 or SWOG-9500 studies [16]-[19]. Cytogenetic studies, centrally reviewed by the SWOG Cytogenetics Committee, were available for most patients. *FLT3*, *NPM1*, and *DNMT3A* molecular studies for the samples have previously been described, and results were obtained from the SWOG database [20]–[22].

### Identification of Novel Splice Variants

The WT-*IRF8* sequence was obtained from the UCSC Human Genome Browser (NM_144701; http://genome.ucsc.edu/cgibin/hgc?hgsid=311274207&c=chr1&o=67632168&t=67725650& g=refGene&i=NM_144701). Qualitative RT/PCR assays were developed to examine the entire coding region of the WT-*IRF8* transcript (Table S1). Primer IRF8.Ex1.F annealed to the 5′ untranslated region (UTR) within exon 1, while primer IRF8.Ex2.F) annealed to exon 2, which contains the start codon. A universal reverse primer (IRF8.Ex9.R) annealed to the 3′ UTR in exon 9 distal to the stop codon. The sequencing primers were scattered approximately every 300–400 Bps along the coding region (Table S1). cDNA was generated using poly T primers, standard reagents, and conditions as previously described. The entire WT-*IRF8* transcript was amplified using High Fidelity Platinum Taq polymerase (Invitrogen, Carlsbad, CA, USA) and conditions provided in Table S1. GeneRacer^TM^ kit (Invitrogen) was utilized for RNA ligase-mediated rapid amplification of 5′ end of IRF8 transcripts using the GeneRacer™ 5′ Primer (forward) and either IRF8.Ex3.R or IRF8.Ex9.GR.R primers (Table S1). Amplified products were then sequenced using the amplification primer set and conditions provided in Table S1.

### Development of Q-RT/PCR Assays for WT-*IRF8* and SV-*IRF8*

**Figure 1:**
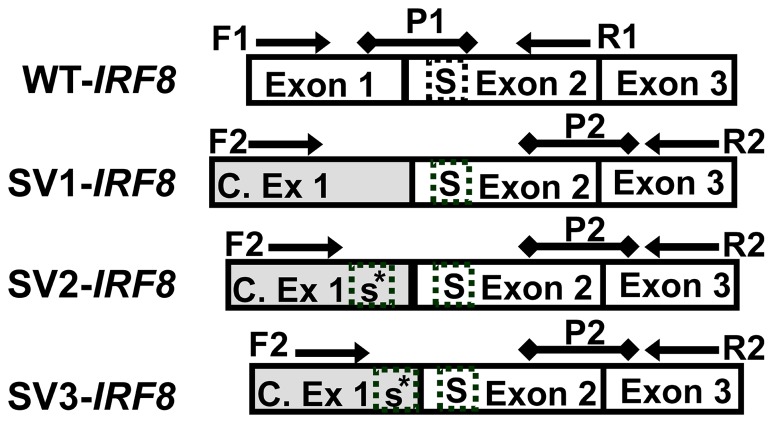
Q-RT/PCR assay design for WT- and SV-*IRF8* transcripts. Arrows represent forward (F) and reverse (R) primers; lines represent probes (P); reference sequence exons are white; cryptic 1^st^ exon (C. Ex 1) within the intron sequences (SV1-3) is shaded. The length of the cryptic 1^st^ exon varies for each of the 3 novel splice variants. The standard start codon of the reference sequence is marked by an “S,” potential alternative start codon within the cryptic exon sequence for SVs is marked by an “s*” for SV2 and SV3.

Based on the consensus sequence of the three SV-*IRF8* transcripts (File S1, *IRF8* Sequences), a forward primer within the cryptic 1^st^ exon was developed to amplify all three SVs. Two probes for quantitative real-time PCR (Q-PCR) assays were developed, P1 and P2. P1 annealed to a unique sequence spanning exons 1 and 2 of the WT-*IRF8* transcript. When coupled with primers F1 and R1, P1 was used to quantify WT-*IRF8* transcripts by Q-PCR. P2 annealed across the junction of exons 2 and 3, and when coupled with primers F2 and R2, P2 was used to quantify the total expression of all three SV-*IRF8* transcripts. Primer and probe sequences are provided in Table S2 and are illustrated in Fig 1.

WT-*IRF8*, SV-*IRF8*, *CD34* and *GUSB* (e.g., housekeeping control) assays were performed in duplicate using cDNA generated from RNA as previously described [23]. Primers, probes, and reagents were mixed and subjected to 45 cycles of amplification using standard thermal cycling conditions on Applied Biosystems 7900HT Fast Real-Time PCR system [23]. All experiments contained appropriate negative and positive controls, including a calibrator sample, i.e., a pool of RNA from peripheral blood mononuclear cells of 10 normal donors. The fold differences were computed using 2^−ΔΔCT^ method, employing *GUSB* expression to correct for RNA integrity and the PB expression as the control sample for computation of fold change as previously described [24].

### Development of Fragment Analysis Assay for Individual SV-*IRF8* Transcripts

A fragment analysis assay was developed utilizing the same primers that were used for Q-RT/PCR SV-*IRF8* assay (i.e., F2 and R2). The forward primer (F2), which annealed to a conserved sequence within the cryptic 1^st^ exon, was fluorescently labeled with FAM. The fragment analysis assay was optimized and performed using cDNA generated from RNA. Primers (FAM-F2 and R2) and standard reagents were mixed and subjected to 35 cycles of amplification (Table S1, *Fragment Analysis*). Analyses were performed using the GeneMapper software as previously described [21].

### Statistical methods

Q-RT/PCR assays for WT-*IRF8*, SV-*IRF8* transcripts, *CD34* and *GUSB* were performed in duplicate. Geometric means of the duplicate fold-change values were used for all statistical analyses (File S2, Additional Statistical Methods). Data regarding patient characteristics and treatment outcomes, including complete response (CR), resistant disease (RD), overall survival (OS), relapse-free survival (RFS), and CR duration were collected and evaluated according to standard SWOG procedures [16]–[19]. OS was measured from the date of the patient's entry into the clinical trial until death from any cause, with observation censored at the date of last contact for patients not known to have died. RFS and CR duration were measured from the date of CR until AML relapse or death from any cause with censoring at the date of last contact for patients with no report of relapse (RFS) or until relapse with censoring at the last contact or death (CR duration). Co-factors for statistical analyses of outcome included *FLT3* internal tandem duplications (*FLT3*-ITD), *NPM1* mutation, *DNMT3A* mutation, age, sex, race, white blood cell (WBC) count, BM and PB blast percentages at study entry, FAB classification, AML onset (i.e., *de novo* vs. secondary), and cytogenetic risk group. Associations between continuous variables were measured by Spearman's rank order correlation coefficient (*ρ*). Comparisons between groups of patients were based on Wilcoxon or Kruskal-Wallis rank sum tests for continuous variables, and on logistic and proportional hazards regression analyses for dichotomous and time-to-event data, respectively. Statistical significance was represented by two-sided p-values (P).

## Results

### Identification of Novel Splice Variants

**Figure 2:**
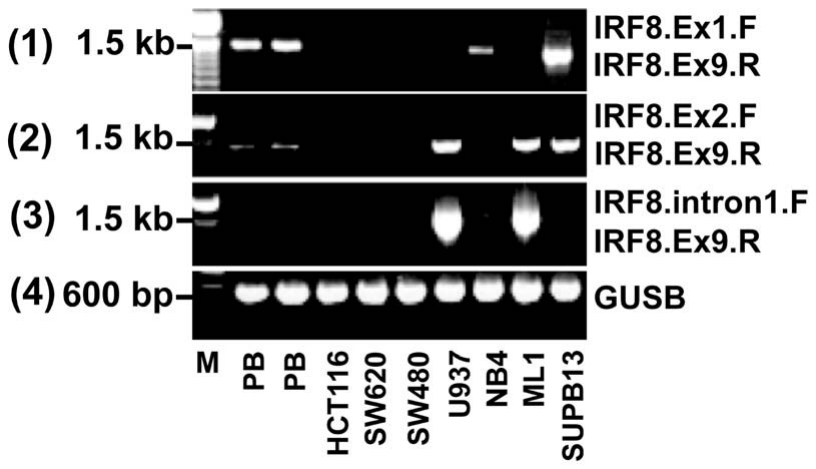
Identification of Novel *IRF8* Splice Variants. **A**. Expression differences in *IRF8* amplification were identified based on whether the forward primer was located in exon 1 (panel 1) or exon 2 (panel 2). These findings led to the hypothesis that exon 1 was not being transcribed in certain cell lines. Novel splice variants were identified using GeneRacer ^TM^ kit and a forward primer was developed to amplify only these splice variants (panel 3). *GUSB* control was used to confirm that cDNA was generated from the intact RNA (panel 4). **B**. Quantitative RT/PCR confirmed the high expression of the SV-*IRF8* (panel 5) in U937 and ML1.

Potential loss of exon 1 was suggested by attempts to amplify the entire sequence of *IRF8* in cell lines. Many of evaluated cell lines showed amplification using Ex2.F/Ex9.R primer set (Fig 2, second panel) but not Ex1.F/Ex9.R (Fig 2, first panel). Using the GeneRacer Kit, three novel splice variants were identified and sequenced. In each case, a cryptic 1^st^ exon originating from within the normal intron 1 region was detected (File S1, *IRF8* Sequences), while exon 1 sequence was missing. A forward primer annealing to all the splice variants was developed and used to confirm that the alternative splice variants retained the entire coding region of *IRF8* (i.e., exons 2–9, Fig 2, panel 3). For two of the splice variants, a potential start codon was identified within the cryptic exon 1 and prior to the normal start codon (Figs 1 and 2, File S1, *IRF8* Sequences). It is unknown if these two splice variants begin translation at the alternative start codon, but preliminary studies demonstrate that the cell lines that primarily transcribe SV-*IRF8* transcripts express an IRF8 protein (File S3, Protein Expression).

### Characterization of patient population

The characteristics of 194 AML patients with available material were compared to 530 subjects from the same SWOG trials who were excluded due to an induction regimen other than Ara-C (Days 1–7) and DNR (Days 1–3), or absence of material for investigation. The 194 included patients had higher WBC counts and blast percentages. In addition, the included patients were younger, more likely to have normal karyotype, and less likely to have deletions of chromosomes 5q and/or 7q (Table 1). After adjusting for these factors, the 194 included patients did not differ significantly in CR or RD rates, RFS, CR duration, or OS from other trial participants.

**Table 1:** Characteristics of patients with *IRF8* data and excluded patients.

		With *IRF8* Data (N = 194)		Without *IRF8* Data (N = 530)		
		N	%	N	%	P b
Sex	Female	86	44%	236	45%	1.00
	Male	108	56%	294	55%	
Race	Asian/P.I.^a^	6	3%	5	1%	0.010
	Black	19	10%	44	8%	
	Nat. Amer.^a^	2	1%	0	0%	
	White	164	86%	478	91%	
	Unknown	3	–	3	–	
Hispanic	Yes	6	3%	16	3%	1.00
Ethnicity?	No	188	97%	511	97%	
	Unknown	0	–	3	–	
Perfor-	0	48	25%	150	29%	0.16
mance	1	97	50%	258	49%	
Status	2	29	15%	83	16%	
	3	20	10%	31	6%	
	Unknown	0	–	8	–	
AML	De novo	88	69%	348	71%	0.67
Onset	Secondary	40	31%	144	29%	
	Unknown	66	–	38	–	
FAB	M1	50	26%	117	22%	0.0023
Class	M2	67	35%	162	31%	
	M4	46	24%	100	19%	
	M5	17	9%	47	9%	
	Other/Unknown	14	7%	104	20%	
*FLT3*-ITD	Present	58	35%	40	26%	0.089
	Absent	107	65%	114	74%	
	Unknown	29	–	376	–	
*NPM1*	Mutant	42	33%	37	31%	0.79
Mutation	WT	86	67%	83	69%	
	Unknown	66	–	410	–	
*DNMT3A*	Mutant	27	20%	23	18%	0.75
Mutation	WT	110	80%	108	82%	
	Unknown	57	–	399	–	

		Median	Min – max	Median	Min – max	P c
Age (yrs)		61	18–88	66	18–86	<0.0001
Marrow blasts (%)		70	5–99	60	0–99	0.0020
WBC (10^9^/L)		32.1	1.2–274	7.3	0.4–298	<0.0001
Peripheral blasts (%)		44	0–99	20	0–99	<0.0001

	With *IRF8* Data (N = 149 d)		Without *IRF8* Data (N = 416 d)		P e
Cytogenetics	N	%	N	%	
Normal	65	44%	140	34%	0.037
Normal + nonclonal	8	5%	10	2%	0.10
t(8;21)	5	3%	9	2%	0.54
inv(16), t(16;16)	8	5%	12	3%	0.19
t(9;11)	3	2%	7	2%	0.73
t(6;9)	0	0%	1	0.2%	1.00
inv(3), t(3;3)	2	1%	2	0.5%	0.28
–5, del(5q)	8	5%	63	15%	0.0014
–7, del(7q)	12	8%	69	17%	0.0096
t(v;11)(v;q23)	2	1%	6	1%	1.00
+8	15	10%	57	14%	0.32
t(15;17)	1	1%	3	1%	1.00
t(9;22)	3	2%	5	1%	0.44
Other	28	19%	86	21%	0.72

Risk Category f	N	%	N	%	P g
Favorable	13	9%	21	5%	0.0019
Intermediate-I	73	49%	150	36%	
Intermediate-II	31	21%	93	22%	
Adverse	32	21%	152	37%	

a P.I.  =  Pacific Islander; Nat. Amer.  =  Native American or Alaskan Native.

b Two-sided p-value based on Fisher's exact test for sex, Hispanic ethnicity, AML onset and mutations; and on Jonckheere-Terpstra test for performance status. P-value based on Pearson's chi-squared test for independence for race (exact calculation) and FAB class.

c Two-sided p-value based on Wilcoxon rank sum test.

d Patients with cytogenetic data only.

e Two-sided p-value based on Fisher's exact test.

f Favorable  =  t(8;21) or inv(16)/t(16;16). Intermediate-I  =  normal, with or without nonclonal abnormality.

Intermediate-II  =  t(9;11) or other abnormality not classified favorable or adverse. Adverse  =  inv(3)/t(3;3), t(6;9), t(v;11)(v;q23), −/del(5q), −7/del(7q).

g P-value based on Pearson's chi-squared test for independence.

### Associations with other clinical and molecular factors

**Figure 3:**
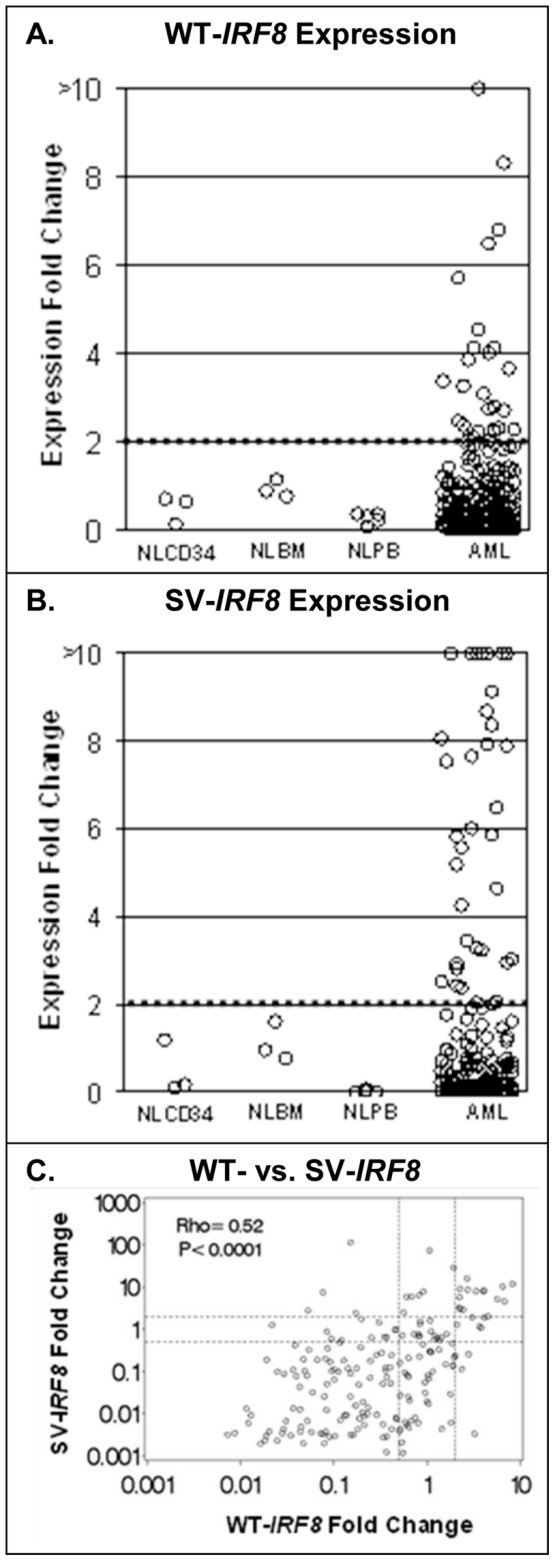
Expression of *IRF8* transcripts in samples from healthy donors and AML patients. **A**. Expression of WT-*IRF8* in samples from normal bone marrow CD34+ cells (NLCD34, N = 3), bone marrow (NLBM, N = 3), peripheral blood (NLPB, N = 5), and AML patients (AML, N = 194). Dashed line marks the expression for a fold change of 2. **B**. Expression of SV-*IRF8* in the samples from the same normal donors and AML patients (AML, N = 192) as in Fig 1A. Dashed line marks the expression for a fold change of 2. **C**. Correlation between expression of WT-*IRF8* (x-axis) and SV-*IRF8* transcripts (y-axis). Dashed lines mark expression for a fold change of 2.

WT-*IRF8* was expressed at low levels in majority of AML patients (range 0.00728–8.317, Fig 3A), but 12% (24/194) displayed a marked increased WT-*IRF8* expression (>2-fold). SV-*IRF8* was also expressed at low levels in most AML patients (range 0.002–115.031, Fig 3B), but, a subgroup of AML patients (26/192, 14%) also displayed >2-fold increase in SV-*IRF8* expression. WT-*IRF8* and SV-*IRF8* expression were significantly correlated (*ρ* = 0.52, P<0.0001); however, many samples with increased SV-*IRF8* expression displayed very low levels of WT-*IRF8* and vice versa (Fig 3C). To determine if mutations within the coding sequence of WT-*IRF8* may be present in samples with increased expression of *IRF8,* ten samples with high expression of both SV- and WT-*IRF8* were sequenced using multiple primers (Table S1). None of these samples harbored mutations within the coding exons of the gene. Increased expression of WT-*IRF8* was significantly associated with FAB M5 phenotype (acute monoblastic or monocytic leukemia, P<0.0001). In addition, increased expression of WT-*IRF8* was modestly associated with higher *CD34* expression (*ρ* = 0.20, P = 0.0046), lower PB blast percentage (*ρ* = −0.18, P = 0.011), and an absence of *FLT3*-ITD (P = 0.027, Tables 2 and 3). WT-*IRF8* expression was not significantly associated with other potential prognostic factors, including age, other molecular biomarkers (*NPM1* or *DNMT3A* mutations) or cytogenetic risk group (Tables 2 and 3).

**Table 2:** Rank order correlation of WT-*IRF8* and SV-*IRF8* expression (fold-change, treated as a continuous variable) with patient characteristics, based on 194 adult patients with previously untreated AML.

	WT-*IRF8* Expression			SV-*IRF8* Expression		
	Pts	*ρ*	P	Pts	*ρ*	P
Age	194	0.12	0.11	192	0.10	0.17
Marrow blasts (%)	180	−0.12	0.11	178	−0.18	0.018
WBC count	194	0.08	0.27	192	−0.07	0.30
Peripheral blasts (%)	188	−0.18	0.011	186	−0.18	0.013
CD34 expression	194	0.20	0.0046	192	0.36	<0.0001

*ρ*  =  Spearman's rank order correlation coefficient; P = 2-tailed p-value.

**Table 3:** Association of WT-*IRF8* expression (fold-change) with patient characteristics, based on 194 adult patients with previously untreated AML.

			Continuous WT-*IRF8* Expression			WT-*IRF8* Expression Categories		
		Pts	Median	Min – Max	P_1_	≤2.0	>2.0	P_2_
Sex	Female	86	0.33	0.01–5.7	1.00	86%	14%	0.55
	Male	108	0.41	0.01–8.3		89%	11%	
Race	Asian/P.I.	6	0.45	0.12–5.7	0.82	83%	17%	0.93
	Black	19	0.30	0.02–3.1		89%	11%	
	Nat. Amer.	2	1.07	0.26–1.9		100%	0%	
	White	164	0.37	0.01–8.3		87%	13%	
	Unknown	3	0.34	0.01–0.4		100%	0%	
Perfor-	0	48	0.20	0.01–3.1	0.055	92%	8%	0.60
mance	1	97	0.42	0.01–6.8		88%	12%	
Status	2	29	0.60	0.02–5.7		86%	14%	
	3	20	0.12	0.02–8.3		80%	20%	
AML	De novo	88	0.37	0.02–8.3	0.088	84%	16%	0.46
Onset	Secondary	40	0.53	0.02–5.7		78%	23%	
	Unknown	66	0.30	0.01–6.5		98%	2%	
FAB	M1	50	0.19	0.01–8.3	<0.0001	90%	10%	0.044
Class	M2	67	0.14	0.01–4.6		93%	7%	
	M4	46	0.64	0.02–4.1		89%	11%	
	M5	17	1.04	0.15–6.5		71%	29%	
	Other*	14	0.93	0.03–6.8		71%	29%	
	M0	7						
	M4/M5	1						
	M4 or M5B	1						
	M6	2						
	M7	1						
	Not Stated	2						
*FLT3*	Positive	58	0.19	0.01–8.3	0.027	91%	9%	0.45
ITD	Negative	107	0.45	0.01–5.7		86%	14%	
	Unknown	29	0.37	0.01–6.8		86%	14%	
*NPM1*	Mutant	42	0.26	0.01–3.7	0.55	95%	5%	0.22
Mutation	WT	86	0.40	0.01–6.5		87%	13%	
	Unknown	66	0.41	0.02–8.3		83%	17%	
*DNMT3A*	Mutant	27	0.73	0.01–2.4	0.090	93%	7%	1.00
Mutation	WT	110	0.30	0.01–6.5		90%	10%	
	Unknown	57	0.41	0.02–8.3		81%	19%	
Cyto-	Favorable	13	0.63	0.05–2.3	0.43	92%	8%	0.53
genetic	Int-Normal	73	0.34	0.01–8.3		88%	12%	
Risk	Int-II	31	0.30	0.04–6.5		84%	16%	
Group	Unfavorable	32	0.57	0.01–4.6		78%	22%	
	Unknown	45	0.29	0.01–6.8		96%	4%	

P.I.  =  Pacific Islander; Nat. Amer.  =  Native American or Alaskan Native; P_1_ =  p-value based on Wilcoxon rank sum test; P_2_ =  p-value from chi-square test for independence (Race, Performance Status, FAB Class, Cytogenetic Risk Group) or Fisher's exact test (Sex, AML Onset, *FLT3*-ITD); unknown categories are excluded from all significance tests.

* =  FAB classifications for 14 patients (as reported by treating institutions based on their local pathology) were combined into “Other” FAB class for statistical purposes due to small numbers of patients in each individual FAB class.

Similar to WT-*IRF8*, increased expression of SV-*IRF8* was associated with higher levels of *CD34* expression and lower PB blast percentage (*ρ* = 0.36, P<0.0001 and *ρ* = −0.18, P = 0.013, respectively). Increased expression of SV-*IRF8* was not significantly associated with M5 FAB phenotype (P = 0.31); however, increased expression of SV-*IRF8* was correlated with lower BM blast percentage (*ρ* = −0.18, P = 0.018) and an absence of *NPM1* mutations (P = 0.039, Tables 2 and 4), with the later finding being particularly true for samples with >2-fold SV-*IRF8* expression (P  = 0.0047). SV-*IRF8* was also not significantly associated with age, other molecular biomarkers (*FLT3*-ITD or *DNMT3A* mutations) or cytogenetic risk groups (Tables 2 and 4).

**Table 4:** Association of SV-*IRF8* expression (fold-change) with patient characteristics, based on 192 adult patients with previously untreated AML.

			Continuous SV-*IRF8* Expression				SV-*IRF8* Expression Categories				
		Pts	Median	Min – Max		P_1_	≤2.0		>2.0		P_2_
Sex	Female	86	0.12	0.002–74.3		0.57	87%		13%		0.83
	Male	106	0.10	0 001–115			86%		14%		
Race	Asian/P.I.	6	0.04	0.001–5.2		0.41	83%		17%		0.68
	Black	18	0.04	0.002–16.1			94%		6%		
	Nat. Amer.	2	0.55	0.46–0.64			100%		0%		
	White	163	0.11	0.001–115			85%		15%		
	Unknown	3	0.05	0.006–0.65			100%		0%		
Perfor-	0	47	0.16	0.001–74.3		0.33	85%		15%		0.28
mance	1	96	0.10	0.002–16.1			90%		10%		
Status	2	29	0.18	0.001–115			76%		24%		
	3	20	0.03	0.002–11.9			90%		10%		
AML	De novo	88	0.11	0.001–115		0.32	83%		17%		0.80
Onset	Secondary	40	0.19	0.001–74.3			80%		20%		
	Unknown	64	0.05	0.002–10.4			95%		5%		
FAB	M1	50	0.04	0.003–115		0.31	88%		12%		0.045
Class	M2	67	0.10	0.002–16.1			88%		12%		
	M4	46	0.21	0.001–8.4			93%		7%		
	M5	16	0.08	0.001–29.1			75%			25%	
	Other*	14	0.10	0.004–8.1			64%			36%	
	M0	7									
	M4/M5	1									
	M4 or M5B	1									
	M6	2									
	M7	1									
	Not Stated	2									
*FLT3*	Positive	57	0.06	0.002–11.9	0.23			88%	12%		0.81
ITD	Negative	106	0.13	0.001–115				86%	14%		
	Unknown	29	0.17	0.001–74.3				86%	14%		
*NPM1*	Mutant	42	0.03	0.001–1.7	0.039			100%	0%		0.0047
Mutation	WT	85	0.11	0.002–115				85%	15%		
	Unknown	65	0.20	0.001–74.3				80%	20%		
*DNMT3A*	Mutant	27	0.11	0.003–5.8	0.65			96%	4%		0.19
Mutation	WT	108	0.09	0.002–115				86%	14%		
	Unknown	57	0.13	0.001–74.3				82%	18%		
Cyto-	Favorable	13	0.14	0.004–9.1	0.40			92%	8%		0.67
genetic	Int-Normal	73	0.09	0.001–16.1				86%	14%		
Risk	Int-II	29	0.28	0.003–115				79%	21%		
Group	Unfavorable	32	0.15	0.002–8.7				88%	13%		
	Unknown	45	0.05	0.002–29.1				89%	11%		

P.I.  =  Pacific Islander; Nat. Amer.  =  Native American or Alaskan Native; P_1_ =  p-value based on Wilcoxon rank sum test; P_2_ =  p-value from chi-square test for independence (Race, Performance Status, Cytogenetic Risk Group) or Fisher's exact test (Sex, AML Onset, *FLT3*-ITD); unknown categories are excluded from all significance tests.

* =  FAB classifications for 14 patients (as reported by treating institutions based on their local pathology) were combined into “Other” FAB class for statistical purposes due to small numbers of patients in each individual FAB class.

### WT-*IRF8* and SV-*IRF8* as Adverse Prognostic Biomarkers

**Figure 4:**
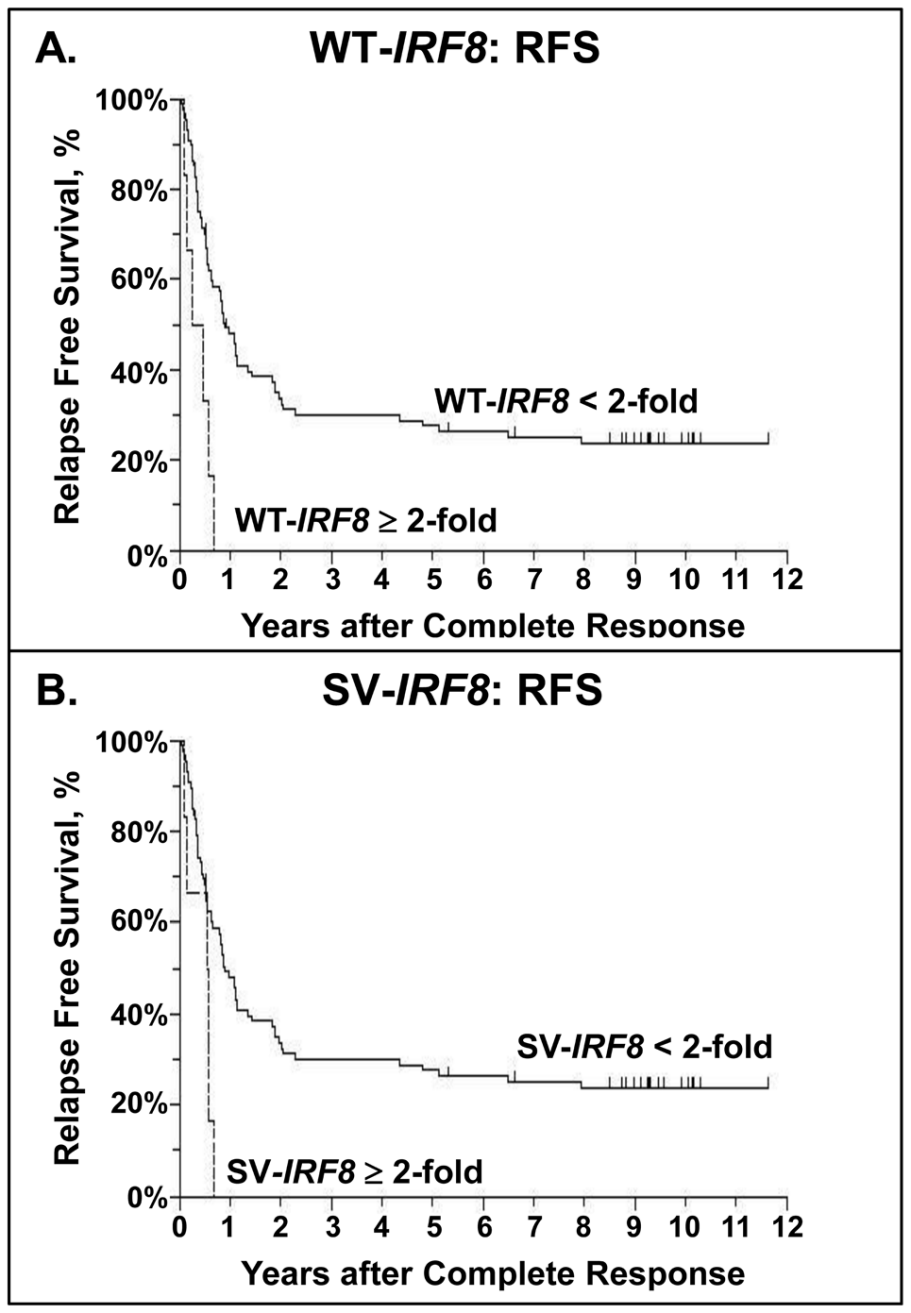
Kaplan-Meier estimates of Relapse-Free Survival for patients who achieved CR. **A**. RFS by WT-*IRF8* (N = 94 patients with CR). **B**. RFS by SV-*IRF8* (N = 92 patients with CR). Tick marks indicate censored observations.

**Figure 5:**
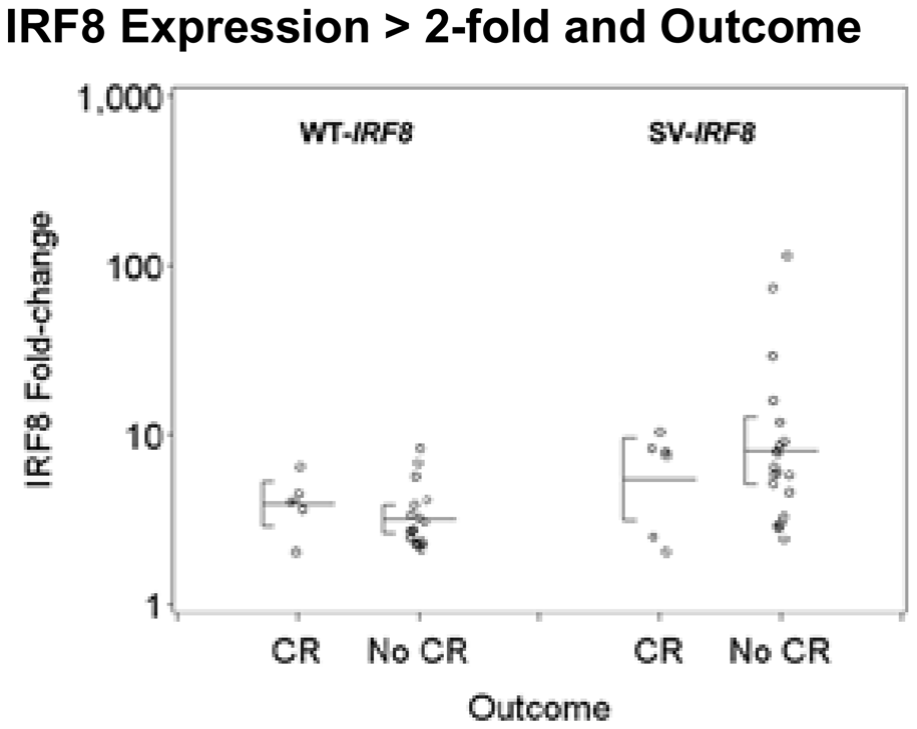
WT- and SV-*IRF8* expression among patients with >2-fold expression, by outcome. Figure shows the variability in WT- and SV-*IRF8* in 24 and 26 patients, respectively, who demonstrate high level of expression, as defined by >2-fold increase. The difference median expression between patients who obtained CR and those who didn't (x-axis) is minimal.

As a continuous variable, WT-*IRF8* expression was not significantly associated with CR, RD, or OS; however, increasing WT-*IRF8* expression was associated with a reduction in RFS (P = 0.010 in univariate analysis, P = 0.019 in multivariate analysis). Examining WT-*IRF8* expression as a dichotomous variable, an increased WT-*IRF8* expression (>2-fold) was associated with a lower CR rate (25%, 6/24, vs. 52%, 88/170, P = 0.012) and a reduced OS as exemplified by an increased hazard ratio (HR) of 1.63 (P = 0.042), but these adverse associations did not retain their significance after adjusting for other prognostic factors (P = 0.33 and P = 0.63, respectively). However, >2-fold expression of WT-*IRF8* was significantly associated with shorter RFS in both univariate (P = 0.0099) and multivariate analyses (P = 0.011). Notably, all patients with >2-fold WT-*IRF8* expression (N = 6) relapsed within 8 months after achieving CR (Fig 4A), with adjusted HR of 3.13 (95% CI 1.30–7.49). These 6 patients were not restricted to a single FAB class (i.e., 1 patient was classified as having FAB M1, another – FAB M2, two patients – FAB M4, and two more – FAB M5). These findings are summarized in Table 5. Since 58 of the 71 RFS events were relapses, the effect of WT-IRF8 on RFS was largely due to its impact on risk of relapse: >2-fold expression was associated with shorter CR duration in univariate (P = 0.0099) and multivariate (P = 0.011) analyses. Within the group of patients with >2-fold WT-*IRF8* expression, patients who obtained a CR and relapsed had WT-*IRF8* levels similar to those who never obtained a CR (Fig 5).

**Table 5:** Treatment outcomes by WT-*IRF8* expression.

				CR,	Complete	Response				RD,	Resistant	Disease	
				Unadjusted		Adjusted				Unadjusted		Adjusted	
	Pts	CR	%CR	OR	95% CI	OR	95% CI	RD	%RD	OR	95% CI	OR	95% CI
≤2-fold	170	88	52%	1	Reference	1	Reference	56	33%	1	Reference	1	Reference
>2-fold	24	6	25%	0.31	0.11–0.78	0.59	0.19–1.66	10	42%	1.45	0.59–3.46	0.68	0.21–2.01
Two groups				P = 0.012		P = 0.33				P = 0.40		P = 0.50	
Trend				P = 0.17		P = 0.98				P = 0.93		P = 0.17	

				OS,	Overall	Survival					RFS,	Relapse		-Free	Survival
				Unadjusted		Adjusted				Unadjusted				Adjusted	
	Pts	Deaths	5-yr	HR	95% CI	HR	95% CI	Events	5-yr	HR			95% CI	HR	95% CI
≤2-fold	170	140	19%	1	Reference	1	Reference	65	28%	1			Reference	1	Reference
>2-fold	24	23	8%	1.63	1.05–2.55	1.12	0.70–1.80	6	0%	3.81			1.60–9.08	3.13	1.30–7.49
Two groups				P = 0.042		P = 0.63				P = 0.0099				P = 0.011	
Trend				P = 0.16		P = 0.61				P = 0.010				P = 0.019	

*OR  =  odds ratio; HR  =  hazard ratio; Events  =  relapses from CR or death from any cause; CI  =  confidence interval.*

*P  =  two-sided p-value from logistic regression (CR, RD) or proportional hazards regression (OS, RFS), treating WT-IRF8 expression as a dichotomous variable (“Two groups”, i.e., >2-fold vs. ≤2-fold) or as a continuous variable (“Trend”).*

*“Adjusted” analyses include the following covariates, which were identified as significant in multivariate analyses:*

*CR: age, CD34 expression (fold-change), and performance status (2–3 vs. 0–1);*

*RD: a*ge, *CD34* expression, and *FLT3*-ITD (present vs. absent);

OS: age, *CD34* expression, and performance status;

RFS: age.

Increasing SV-*IRF8* expression as a continuous variable was associated with lower CR rate (P = 0.0023), higher RD (P = 0.028) and lower OS (P = 0.051), but these associations did not retain their significance after adjusting for other prognostic factors. Similar to WT-*IRF8*, increasing SV-*IRF8* expression was associated with reduced RFS (P = 0.026), which remained significant after adjusting for other prognostic factors (P = 0.021). Furthermore, as a dichotomous variable, >2-fold SV-*IRF8* expression was also significantly associated with reduced RFS in both unadjusted (P = 0.029) and adjusted (P = 0.017) analyses (Fig 4B). Patients with >2-fold SV-*IRF8* had an adjusted HR for RFS of 2.89 (95% CI 1.21–6.89). These findings are summarized in Table 6. As with WT-*IRF8*, the effect of SV-*IRF8* expression was primarily on risk of relapse: >2-fold expression was associated with shorter CR duration in univariate (P = 0.043) and multivariate (P = 0.027) analyses. Similarly, patients with >2-fold SV-*IRF8* expression who obtained a CR and relapsed did not display a markedly different median expression levels than patients who never obtained a CR (Fig 5).

**Table 6:** Treatment outcomes by SV-*IRF8* expression.

				CR,	Complete	Response				RD,	Resistant	Disease	
				Unadjusted		Adjusted				Unadjusted		Adjusted	
	Pts	CR	%CR	OR	95% CI	OR	95% CI	RD	%RD	OR	95% CI	OR	95% CI
≤2-fold	166	86	52%	1	Reference	1	Reference	52	31%	1	Reference	1	Reference
>2-fold	26	6	23%	0.28	0.10–0.69	0.55	0.18–1.56	14	54%	2.56	1.11–6.00	1.51	0.52–4.33
Two groups				P = 0.0051		P = 0.27				P = 0.028		P = 0.44	
Trend				P = 0.0023		P = 0.27				P = 0.028		P = 0.49	

				OS,	Overall	Survival				RFS,	Relapse	-Free	Survival
				Unadjusted		Adjusted				Unadjusted		Adjusted	
	Pts	Deaths	5-yr	HR	95% CI	HR	95% CI	Events	5-yr	HR	95% CI	HR	95% CI
≤2-fold	166	138	19%	1	Reference	1	Reference	64	28%	1	Reference	1	Reference
>2-fold	26	24	12%	1.41	0.91–2.19	1.01	0.63–1.62	6	0%	3.01	1.26–7.18	2.89	1.21–6.89
Two groups				P = .14		P = 0.98				P = 0.029		P = 0.017	
Trend				P = 0.051		P = 0.26		41		P = 0.026		P = 0.021	

OR  =  odds ratio; HR  =  hazard ratio; Events  =  relapses from CR or death from any cause; CI  =  confidence interval.

P  =  two-sided p-value from logistic regression (CR, RD) or proportional hazards regression (OS, RFS), treating SV-*IRF8* expression as a dichotomous variable (“Two groups”, i.e., >2-fold vs. ≤2-fold) or as a continuous variable (“Trend”).

“Adjusted” analyses include the following covariates, which were identified as significant in multivariate analyses.

CR: age, *CD34* expression (fold-change), and performance status (2–3 vs. 0–1);

RD: *CD34* expression, and *FLT3*-ITD (present vs. absent);

OS: age, *CD34* expression, and performance status;

RFS: age.

Multivariate models were used to test whether the apparent effect of *IRF8* on RFS might be due to the association of *IRF8* with FAB. These analyses were limited to patients with FAB M1, M2, M4 or M5 (combined as M4 or M5 vs. M1 or M2 to enhance statistical power); the two patients with “Other” FAB due to “M4/M5” or “M4 or M5B” were included in the “M4 or M5” group. Although patients with >2-fold WT-*IRF8* expression accounted for a slightly higher proportion of the M4 or M5 group (4/40 = 10%) compared to M1 or M2 (2/50 = 4%), this difference was small. This trend was also observed in the group of patients with >2-fold SV-*IRF8* expression: 3/38 = 7.9% for M4 or M5 vs. 3/50 = 6% for M1 or M2. In multivariate proportional hazards regression analyses incorporating both FAB and *IRF8* expression, *IRF8* retained its significant effects on RFS, while FAB (M4 or M5 vs. M1 or M2) was not significant (Table S3).

### Correlation between individual SV-*IRF8* transcripts and clinical outcomes

Fragment analyses data were available for 165 of the 194 patients. The most abundant dominant transcript variant was SV1-*IRF8* (125/165, 76%), followed by SV2-*IRF8* (28/125, 17%) and SV3-*IRF8* (12/165, 12%). SV1-*IRF8*, as a continuous variable, was associated with higher expression of WT-*IRF8* (P = 0.0001), while there were no significant associations between individual splice variants and SV-*IRF8* expression (Table S4). CD34 expression was somewhat higher in patients with dominant SV2-*IRF8* (P = 0.0091, Table S4).

**Figure 6:**
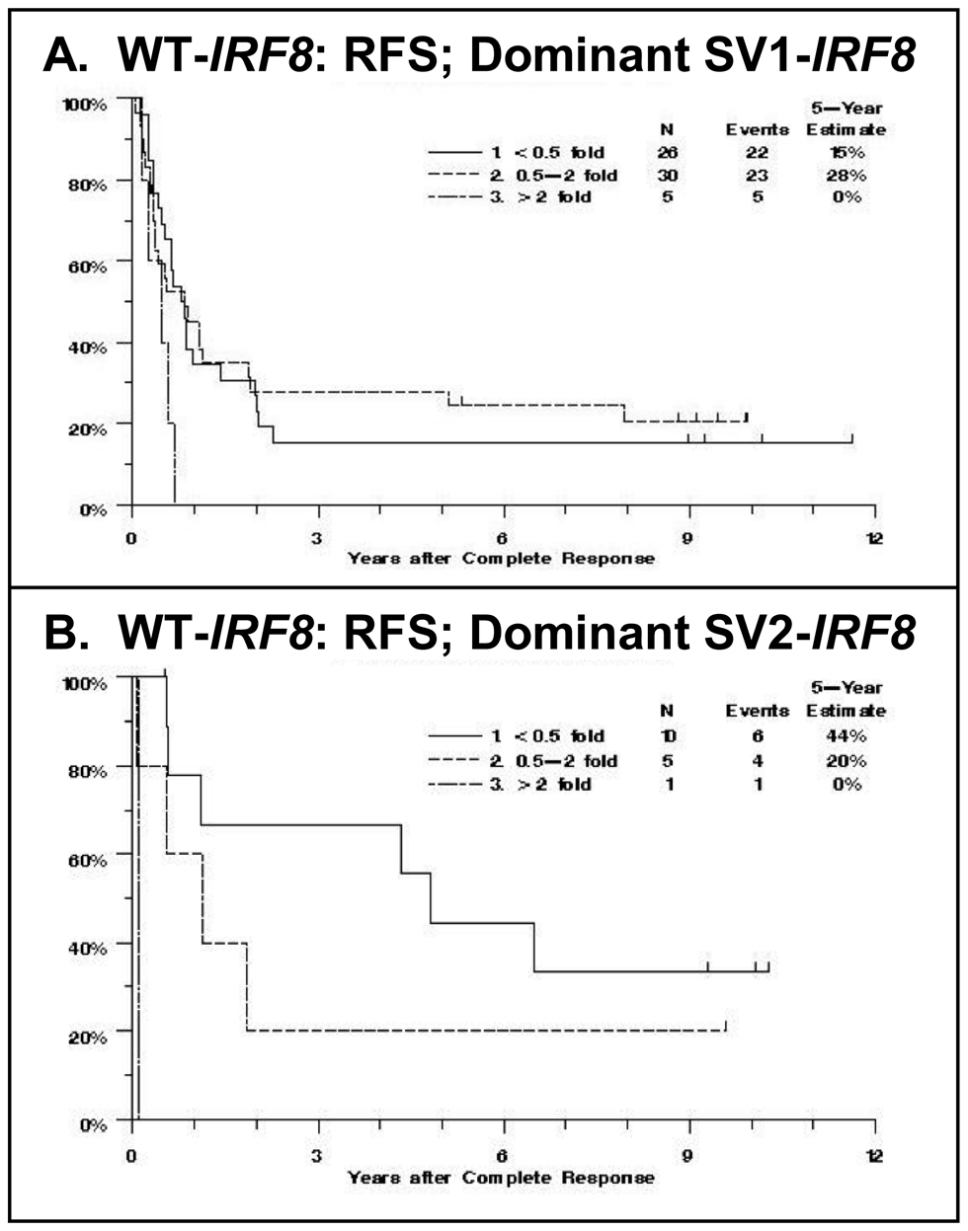
Kaplan-Meier estimates of Relapse-free survival by WT-*IRF8* expression and dominant *IRF8* splice variant (SV1 or SV2). **A**. RFS by WT-*IRF8* expression stratified by the dominant SV1 transcript (N = 61 patients with CR). B. RFS by SV-*IRF8* expression stratified by the dominant SV2 transcript (N = 16 patients with CR). Tick marks indicate censored observations.

Variations in clinical outcomes for the three dominant SV transcripts were not statistically significant (CR = 0.38; RD P = 0.25; OS P = 0.077; RFS P = 0.32, Table S5). Additional analyses were performed to test whether the above-described prognostic effects for WT-*IRF8* or SV-*IRF8* varied significantly between the SV1- and SV2–dominant patients; the SV3 group was excluded due to its small size. In multivariate analyses, adjusting for other significant prognostic factors, there were no significant interactions between the dominant SV transcript and SV-*IRF8* expression for the outcomes of CR, RD, RFS, or OS (Table S6). Likewise, there were no significant interactions between the dominant SV transcript and WT-*IRF8* expression for the outcomes of CR, RD, or OS. There was a modestly significant interaction between the dominant SV transcript and the effect of WT-*IRF8* on RFS, with the impact of WT-*IRF8* expression on RFS being greater for SV1 compared to SV2 (P = 0.043 for continuous expression and P = 0.036 for >2–fold expression; Fig 6). However, due to the small number of SV2-dominant patients in this analysis (N = 16), this result must be interpreted with caution.

## Discussion

Although current prognostic biomarkers (e.g., *FLT3*-ITD, *NPM1* mutations, etc.) have been helpful in risk-stratifying patients with AML, these molecular biomarkers remain somewhat unsatisfactory for accurately predicting clinical outcomes for a subset of patients with AML. Given the molecular heterogeneity and complexity of AML, it is unlikely that a single or very restricted number of prognostic biomarkers will ever display the predictive power necessary to accurately predict clinical outcomes for all AML patients. Therefore, there is a need to identify additional molecular biomarkers that can be combined with other known prognostic factors to more precisely predict clinical outcomes for patients with AML. Previous studies have not examined the prognostic significance of *IRF8* transcripts [10]–[14]. Our results indicate that WT-*IRF8* and SV-*IRF8* transcripts are adverse prognostic biomarkers for predicting RFS in adult patients with AML patients. Moreover, the prognostic significance of these biomarkers was independent of other known prognostic factors. For example, after adjusting for potential associated prognostic factors (e.g., *NPM1*, FAB, etc.), increased expression of WT-*IRF8* and SV-*IRF8* transcripts remained significantly associated with an inferior RFS. Given the independent nature of these biomarkers, *IRF8* transcripts, therefore, may be combined with other prognostic factors (i.e., molecular and clinical) to develop a more comprehensive risk-stratification tool for patients with AML.

Since loss of IRF8 activity has been associated with dysfunctional hematopoiesis and leukemogenesis, it was somewhat unexpected that increased WT-*IRF8* expression was associated with an overall worse prognosis. However, just as a loss of *IRF8* expression disrupts hematopoiesis [25], constitutive or over-expression of *IRF8* may also have a detrimental effect on normal hematopoiesis. Certainly, forced over-expression of IRF8 blocks neutrophil differentiation and promotes the development of macrophages, monocytes and dendritic cells [5], [26], [27]. Although the current correlative studies were not designed to precisely characterize the functional changes associated with increased *IRF8* expression, increased WT-*IRF8* expression was significantly associated with FAB M5 AML (i.e., monocytic leukemia), which is consistent with previous *ex vivo* studies examining the functional impact of over-expression of WT-*IRF8* [5], [26], [27]. Furthermore, if increased *IRF8* expression causes the leukemic blasts to harbor aberrant dendritic-like qualities, these functional changes may explain some of adverse prognostic impact for increased WT-*IRF8*, given that classic dendritic cell leukemia/neoplasms have a particularly unfavorable prognosis [28].

With respect to SV-*IRF8*, it remains uncertain if SV-*IRF8* transcripts are translated into proteins or may be surrogate biomarkers for some other unfavorable biological state. If the splice variants are translated into structurally different proteins than WT-*IRF8*, these structural differences may lead to functional changes, which could explain the negative clinical impact of the aberrant splice variants. This may be especially true if the abnormal protein(s) abrogate or disrupt the function of the WT protein, resulting in a dominant-negative phenotype. Previous studies have shown that expression of WT-*IRF8* transcript is normally regulated by the methylation status of its promoter, such that hypermethylation of the promoter typically down regulates the expression of the WT-*IRF8* transcript [29]. However, we have found that aberrant hypermethylation of the normal *IRF8* promoter may also promote the expression of the SV-*IRF8* transcripts (unpublished data). Hence, expression of the *IRF8* splice variants may be a surrogate biomarker for global hypermethylation of the genome and the potential adverse consequences of this hypermethylation. Likewise, hypermethylation of normal *IRF8* promoter may interfere with the normal regulatory processes controlling *IRF8* transcription. In this case, even if SV-*IRF8* transcripts code for the wild-type protein, the expression of the IRF8 protein may be at inappropriate times or levels, and as such, may lead to cellular dysfunction.

In conclusion, patients with increased expression of WT-*IRF8* or SV-*IRF8* transcripts have significantly shorter durations of remission and RFS. Studies are underway to examine the functional impact of increased WT-*IRF8* expression on chemotherapy sensitivity and the coding potential of the SV-*IRF8* transcripts. Furthermore, larger correlative studies examining more diverse populations of patients with AML and other hematopoietic malignancies are planned. These future correlative investigations will examine *IRF8* transcripts and other molecular biomarkers across broad range of different therapies and ages, including pediatric subjects.

## Supporting Information

Table S1 **Qualitative PCR primers.**
(PDF)

Table S2 **Quantitative RT/PCR primers and Commercial Assays.**
(PDF)

Table S3 **The Effect of** ***IRF8*** **Expression on RFS and associations with FAB.**
(PDF)

Table S4 **Association of** ***IRF8*** **and CD34 expression (fold-change) with dominant** ***IRF8*** **splice variant, based on 165 adult patients with previously untreated AML.**
(PDF)

Table S5 **Association of treatment outcomes with dominant** ***IRF8*** **splice variant, based on 165 adult patients with previously untreated AML.**
(PDF)

Table S6 **Summary of multivariate analyses testing whether prognostic effects of WT- or SV-** ***IRF8*** **differ between SV1- and SV2-** ***IRF8*** **transcripts.**
(PDF)

File S1 ***IRF8*** **Sequences.**
(PDF)

File S2 **Additional Statistical Methods.**
(PDF)

File S3 ***IRF8*** **Protein.**
(PDF)
