# Supplementary material for: The Prognostic Significance of IRF8 Transcripts in Adult Patients with Acute Myeloid Leukemia
Source: PLoS One. 2013 Aug 14;8(8):e70812. doi: 10.1371/journal.pone.0070812 (PMC3743845; doi:10.1371/journal.pone.0070812)
Supplement: Table S1 — Qualitative PCR primers. (PDF) [file pone.0070812.s001.pdf]

Supporting Information, Table S1: Qualitative PCR primers.

Primer Name	Location / Application	Sequence/
IRF8.Ex1.F	5' UTR / amplification, sequencing	GCAGCAAGCGTGGGAAC
IRF8.Ex2.F	Exon 2 / amplification, sequencing	CAGGATGTGTGACCGGAATG
IRF8.Ex9.R	3' UTR / amplification, sequencing	GATGCGGGCCACTGTAAC
IRF8.Ex4.5.F	exons 4, 5 junction / sequencing	GATCAAGGAGCCTTCTGTGGAC
IRF8.Ex7.8.R	Exons 7, 8 junction / sequencing	CATCTGGGAGAATGCTGAATG
IRF8.Ex9.GR.R	3' UTR / GeneRacer amplification, sequencing	CAGAGGGATCCACATTCTTTAATCATGATG
IRF8.Ex3.R	Exon 3 / sequencing, GeneRacer amplification, TAQman*, and Fragment Analysis	CAAGTGGCTGGTTCAGCTTTG
IRF8.intron1.F	Cryptic Exon / amplification, sequencing, TAQman*, and Fragment Analysis (FAM-labeled)	CACTCAGGGCTGTGAGGTCAT

*** Same primer as listed in Supplemental Table S2**

Amplification Conditions for:

Qualitative PCR

95°C for 5 min
95°C for 1 min
60°C for 30 sec x40 cycles
72°C for 4.5 min
72°C for 7 min
4°C hold

Fragment Analysis:

95°C for 5 min
95°C for 1 min
63°C for 30 sec x35 cycles
72°C for 2 min
72°C for 7 min
4°C hold
