# Supplementary material for: The Prognostic Significance of IRF8 Transcripts in Adult Patients with Acute Myeloid Leukemia
Source: PLoS One. 2013 Aug 14;8(8):e70812. doi: 10.1371/journal.pone.0070812 (PMC3743845; doi:10.1371/journal.pone.0070812)
Supplement: Table S2 — Quantitative RT/PCR primers and Commercial Assays. (PDF) [file pone.0070812.s002.pdf]

Supporting Information Table S2: Quantitative RT/PCR primers and Commercial Assays

Assay	Primer/Probe Name	Sequence/Vendor ID (Vendor)
RefSeq-*IRF8*	IRF8.ex1.F (F1)	CAGCAAGCGTGGGAACG
	IRF8.ex2.R (R1)	CTCGATCAGCCACTGTCGAA
	IRF8.ex1.2.probe (P1)	CGGCAGGATG TGTGACCGGA ATG
SV-*IRF8*	IRF8.CE.F (F2)	CACTCAGGGCTGTGAGGTCAT
	IRF8.ex3.R (R2)	C AAGTGGCTGG TTCAGCTTTG
	IRF8.ex2.3.probe (P2)	TGCCT CCATTTTTAA GGCCTGGGC
CD34	Commercial assay	Applied Biosystems Hs00156373_m1
GUSB	Commercial assay	Applied Biosystems 4333767F
