# Supplementary material for: The Prognostic Significance of IRF8 Transcripts in Adult Patients with Acute Myeloid Leukemia
Source: PLoS One. 2013 Aug 14;8(8):e70812. doi: 10.1371/journal.pone.0070812 (PMC3743845; doi:10.1371/journal.pone.0070812)
Supplement: Table S3 — The Effect of IRF8 Expression on RFS and associations with FAB. (PDF) [file pone.0070812.s003.pdf]

Supporting Information, Table S3. The Effect of *IRF8* expression on RFS and associations with FAB.

	WT-*IRF8*		SV-*IRF8*		
FAB Class	>2-fold	≤2-fold	Unknown	>2-f7old	≤2-fold
M1 or M2	48	2	0	47	3
M4 or M5	36	4	2	35	3

	Model with WT-*IRF8* and FAB			Model with WT-*IRF8* and FAB		
	HR	95% CI	P_1	HR	95% CI	P_1
WT-*IRF8* (>2 vs.≤2)	3.75	1.41-8.38	0.0032	---	---	---
SV-*IRF8* (>2 vs.≤2)	---	---	---	3.03	0.14-6.77	0.013
M4 or M5 vs. M1 or M2	1.19	0.73-1.92	0.49	1.23	0.76-1.99	0.40

P_1 = p-value based on proportional hazards regression model.
