# Supplementary material for: The Prognostic Significance of IRF8 Transcripts in Adult Patients with Acute Myeloid Leukemia
Source: PLoS One. 2013 Aug 14;8(8):e70812. doi: 10.1371/journal.pone.0070812 (PMC3743845; doi:10.1371/journal.pone.0070812)
Supplement: Table S4 — Association of IRF8 and CD34 expression (fold-change) with dominant IRF8 splice variant, based on 165 adult patients with previously untreated AML. (PDF) [file pone.0070812.s004.pdf]

**Supporting Information Table S4. Association of *IRF8* and CD34 expression (fold-change) with dominant IRF8 splice variant, based on 165 adult patients with previously untreated AML.**

| Dominant | | Continuous RefSeq-*IRF8* Expression | | | RefSeq-*IRF8* Expression Categories | | |
|---|---|---|---|---|---|---|---|
| Variant | Pts | Median | Min - Max | $P_1$ | ≤2.0 | >2.0 | $P_2$ |
| SV1 | 125 | 0.62 | 0.21 – 8.3 | 0.0001 | 82% | 18% | 0.12 |
| SV2 | 28 | 0.17 | 0.01 – 6.5 | | 93% | 7% | |
| SV3 | 12 | 0.13 | 0.02 – 1.0 | | 100% | 0% | |

| Dominant | | Continuous SV-*IRF8* Expression | | | SV-*IRF8* Expression Categories | | |
|---|---|---|---|---|---|---|---|
| Variant | Pts | Median | Min - Max | $P_1$ | ≤2.0 | >2.0 | $P_2$ |
| SV1 | 125 | 0.14 | 0.001 – 115 | 0.21 | 86% | 14% | 0.11 |
| SV2 | 28 | 0.44 | 0.002 – 29.1 | | 71% | 29% | |
| SV3 | 12 | 0.16 | 0.002 – 5.8 | | 92% | 8% | |

| Dominant | | Continuous CD34 Expression | | | CD34 Expression Categories | | |
|---|---|---|---|---|---|---|---|
| Variant | Pts | Median | Min - Max | $P_1$ | ≤2.0 | >2.0 | $P_2$ |
| SV1 | 125 | 149 | 0.01 – 5424 | 0.0091 | 17% | 83% | 0.065 |
| SV2 | 28 | 467 | 7.1 – 2002 | | 0% | 100% | |
| SV3 | 12 | 145 | 0.4 – 1673 | | 17% | 83% | |

$P_1$ = p-value based on Kruskal-Wallis test; $P_2$ = p-value from chi-square test for independence
