# Supplementary material for: The Prognostic Significance of IRF8 Transcripts in Adult Patients with Acute Myeloid Leukemia
Source: PLoS One. 2013 Aug 14;8(8):e70812. doi: 10.1371/journal.pone.0070812 (PMC3743845; doi:10.1371/journal.pone.0070812)
Supplement: Table S5 — Association of treatment outcomes with dominant IRF8 splice variant, based on 165 adult patients with previously untreated AML. (PDF) [file pone.0070812.s005.pdf]

Supporting Information Table S5. Association of treatment outcomes with dominant *IRF8* splice variant, based on 165 adult patients with previously untreated AML.

Dominant Variant	Pts	Complete Response CR	%	P_1	Resistant Disease RD	%	P_1
SV1	125	61	49%	0.38	43	34%	0.25
SV2	28	16	57%	[0.21]	9	32%	[0.092]
SV3	12	4	33%		7	58%	

Dominant Variant	Pts	Overall Survival Deaths	HR	P_1	Relapse-Free Survival Events	HR	P_1
SV1	125	110	1.00	0.077	50	1.00	0.32
SV2	28	20	0.60	[0.047]	11	0.62	[0.51]
SV3	12	10	1.01		3	0.78	

P_1 = p-value based on univariate logistic (CR, RD) or proportional hazards (OS, RFS) regression model (p-value based on multivariate model is shown in brackets).
Analysis of RFS is limited to patients who achieved CR, events include relapse of AML or death from any cause.
