# Supplementary material for: The Prognostic Significance of IRF8 Transcripts in Adult Patients with Acute Myeloid Leukemia
Source: PLoS One. 2013 Aug 14;8(8):e70812. doi: 10.1371/journal.pone.0070812 (PMC3743845; doi:10.1371/journal.pone.0070812)
Supplement: Table S6 — Summary of multivariate analyses testing whether prognostic effects of WT- or SV- IRF8 differ between SV1- and SV2- IRF8 transcripts. (PDF) [file pone.0070812.s006.pdf]

Supporting Information Table S6. Summary of multivariate analyses testing whether prognostic effects of WT- or SV-*IRF8* differ between SV1- and SV2-*IRF8* transcripts.

	Interaction of Dominant Peak with WT-*IRF8*		Interaction of Dominant Peak with SV-*IRF8*	
Treatment Outcome	Trend	>2-fold vs ≤2-fold	Trend	>2-fold vs ≤2-fold
Complete Response	0.80	0.62	0.90	0.47
Resistant Disease	0.68	0.43	0.42	0.53
Overall Survival	0.66	0.44	0.21	0.13
Relapse-Free Survival	0.043	0.036	0.088	0.26

This table shows likelihood ratio test p-values for testing whether the effect of WT-*IRF8* or SV-*IRF8* expression on treatment outcomes differs between patients with SV1 versus SV2, based on multivariate logistic regression (CR, RD) or proportional hazards regression (OS, RFS) models.
