# Supplementary material for: The Prognostic Significance of IRF8 Transcripts in Adult Patients with Acute Myeloid Leukemia
Source: PLoS One. 2013 Aug 14;8(8):e70812. doi: 10.1371/journal.pone.0070812 (PMC3743845; doi:10.1371/journal.pone.0070812)
Supplement: File S1 — IRF8 Sequences. (PDF) [file pone.0070812.s007.pdf]

Supporting Information, File S1, *IRF8* Sequences:
Reference Sequence *IRF8* (exons 1 and 2)

Nucleotide Sequence

AGCGCGGCAG CAAGCGTGGG AACGCGGGCG GCGAGACGGC GGCAGGACGG
CGGCA**GG**ATG TGTGACCGGA ATGGTGGTCG GCGGCTTCGA CAGTGGCTGA
TCGAGCAGAT TGACAGTAGC ATGTATCCAG GACTGATTTG GGAGAATGAG
GAGAAGAGCA TGTTCCGGAT CCCTTGGAAA CACGCTGGCA AGCAAGATTA
TAATCAGGAA GTGGATGCCT CCATTTTTAA **G**…

Protein Sequence

MCDRNGGRRLRQWLIEQIDSSMYPGLIWENEEKSMFRIPWK…

Splice Variant 1 (SV1) *IRF8* (exons 1 and 2)

Nucleotide Sequence

AGTTTGCACT CAGGGCTGTG AGGTCATGGA GGCCAGCATT GCCTTCTCAT
GGCAGGTGTC CCGGAGTCCC TGAATCTGTG GGTTTCCCCC AAGCCAGCAC
CTTTGCTGCA AACCTCTGAG TTTCCTGTTA GCAGTTTTTG GGTTGCTGTG
ATGAATGAGA CAATATCCGT AATATCACAG CGTGTATTTC TGTCTTTCCA
A**GG**ATGTGTG ACCGGAATGG TGGTCGGCGG CTTCGACAGT GGCTGATCGA
GCAGATTGAC AGTAGCATGT ATCCAGGACT GATTTGGGAG AATGAGGAGA
AGAGCATGTT CCGGATCCCT TGGAAACACG CTGGCAAGCA AGATTATAAT
CAGGAAGTGG ATGCCTCCAT TTTTAA**G**…

Protein Sequence

MCDRNGGRRLRQWLIEQIDSSMYPGLIWENEEKSMFRIPWK…

Splice Variant 2 (SV1) *IRF8* (exons 1 and 2)

Nucleotide Sequence

AGTTTGCACT CAGGGCTGTG AGGTCATGGA GGCCAGCATT GCCTTCTCAT
GGCAGGTGTC CCGGAGTCCC TGAATC**TG**AT GTGTGACCGG AATGGTGGTC
GGCGGCTTCG ACAGTGGCTG ATCGAGCAGA TTGACAGTAG CATGTATCCA
GGACTGATTT GGGAGAATGA GGAGAAGAGC ATGTTCCGGA TCCCTTGGAA
ACACGCTGGC AAGCAAGATT ATAATCAGGA AGTGGATGCC TCCATTTTTA
A**G**…

Protein Sequence

MAGVPESLNL**M**CDRNGGRRLRQWLIEQIDSSMYPGLIWENEEKSMFRIPWK…

Splice Variant 3 (SV1) *IRF8* (exons 1 and 2)

Nucleotide Sequence

AGTTTGCACT CAGGGCTGTG AGGTCATGGA GGCCAGCATT GCCTTCTCAT 50
GGATGTGTGA CCGGAATGGT GGTCGGCGGC TTCGACAGTG GCTGATCGAG 100
CAGATTGACA GTAGCATGTA TCCAGGACTG ATTTGGGAGA ATGAGGAGAA 150
GAGCATGTTC CGGATCCCTT GGAAACACGC TGGCAAGCAA GATTATAATC 200
AGGAAGTGGA TGCCTCCATT TTTAA **G**…

Protein Sequence

MEASIAFSW**M**CDRNGGRRLRQWLIEQIDSSMYPGLIWENEEKSMFRIPWK…
