# Supplementary material for: The Prognostic Significance of IRF8 Transcripts in Adult Patients with Acute Myeloid Leukemia
Source: PLoS One. 2013 Aug 14;8(8):e70812. doi: 10.1371/journal.pone.0070812 (PMC3743845; doi:10.1371/journal.pone.0070812)
Supplement: File S2 — Additional Statistical Methods. (PDF) [file pone.0070812.s008.pdf]

Supporting Information, File S2, Additional Statistical Methods

Calculation of geometric mean values for SV-*IRF8*.

Because SV-*IRF8* levels were quite low for many patients, a substantial number of specimens had no amplification after 45 cycles. The SV-*IRF8* fold-change values for these specimens were treated as left-censored at the value corresponding to Ct=45.

The geometric mean was used to define a single measurement of SV-*IRF8* expression from the duplicate values available for each patient. Let F_{ij} denote the SV-IRF8 fold-change value from replicate *j* =1, 2 for patient *i* = 1, …, *N*. To account for the censored values, each patient's geometric mean was estimated by fitting a mixed model in which the log-transformed fold-change value is written

$$ln(F_{ij}) = \beta + \gamma_i + \varepsilon_{ij} ,$$

where β is the overall mean, and the random components γ_i ~ i.i.d. N(0,σ_B^2) represent the variation between patients, while ε_{ij} ~ i.i.d. N(0,σ_W^2) represent the variation between each patient's replicate specimens, where ε_{ij} and γ_i are independent for all *i* and *j*.

Maximum likelihood estimates $\hat{\beta}$ and $\hat{\gamma}_1, \ldots, \hat{\gamma}_N$ were calculated using SAS 9.2 PROC NLMIXED, and the geometric mean for each patient was then estimated by $\hat{\beta} + \hat{\gamma}_i$. For patients with uncensored values for both replicates, this estimate is simply the usual sample geometric mean $\sqrt{F_{i1} \times F_{i2}}$.

SV-*IRF8* expression data were actually available for a total of 242 specimens, including the 194 from SWOG patients described included in the analysis, and the mixed model analysis was based on all available data from the 242 specimens. Availability of SV-*IRF8* fold-change data are summarized in the following table for all 242 patients and for the 194 patients included in this analysis. SV-*IRF8* data were not available two of the 194 patients. Of the remaining 192 patients, two had successful RT-PCR for only one of their two duplicate specimens.

No. of SV-IRF8 fold-change values obtained	Uncensored / Censored	All Patients	Included Patients
0	NA	2	2
1	1 / 0	2	1
1	0 / 1	1	1
2	2 / 0	126	97
2	1 /1	40	34
2	0 / 2	71	59
Total		242	194
