# Supplementary material for: The Prognostic Significance of IRF8 Transcripts in Adult Patients with Acute Myeloid Leukemia
Source: PLoS One. 2013 Aug 14;8(8):e70812. doi: 10.1371/journal.pone.0070812 (PMC3743845; doi:10.1371/journal.pone.0070812)
Supplement: File S3 — IRF8 Protein. (PDF) [file pone.0070812.s009.pdf]

Supporting Information, File S3, IRF8 Protein:

Cell were lysed in 0.5% Triton X-100 Lysis Buffer containing 1M Tris (pH7.5), 0.5 M EDTA and 1X Protease inhibitor cocktail (Roche Cat#1183617000) and centrifuged at maximum speed at 4°C for 10 minutes. The supernatant was collected and quantified using DC Protein Assay (Bio Rad, Hercules, CA). Protein (25 μg) was resolved on a NuPAGE 4-12% Bis-Tris polyacrylamide gel (Invitrogen, Carlsbad, CA) per standard western blot protocols. IRF8 was probed with goat polyclonal antibody (1:100, C-19, Santa Cruz Biotechnology, Dallas, TX) and the HRP-conjugated rabit-anti-goat IgG secondary antibody (1:5000, Santa Cruz Biotechnology). The protein was revealed using ECL detection reagents (GE Healthcare Life Technologies, Pittsburgh, PA)

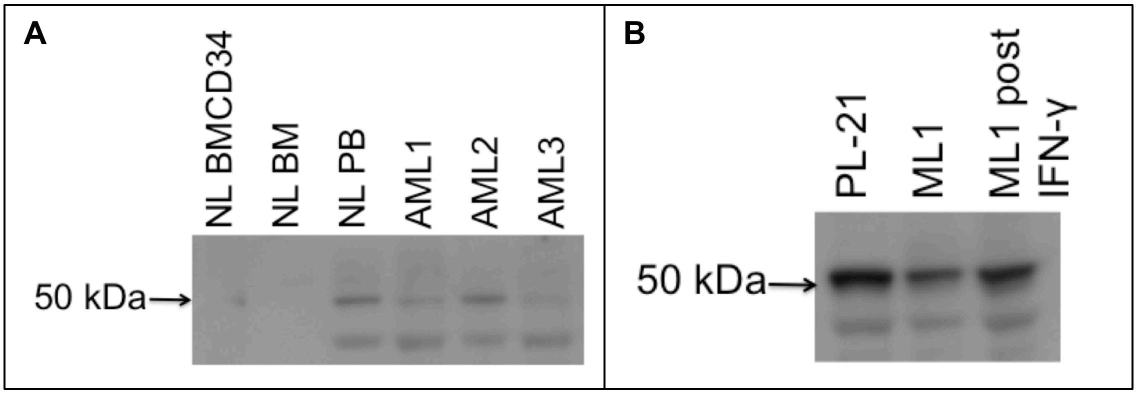

Figure shows the IRF8 protein expressed in bone marrow CD34+ cells (NL BMCD34), bone marrow (NL BM), peripheral blood (NL PB) of normal controls and three AML patients (AML1, 2, and 3). The Expression o
